# High-quality permanent draft genome sequence of the *Mimosa asperata* - nodulating *Cupriavidus* sp. strain AMP6

**DOI:** 10.1186/s40793-015-0074-1

**Published:** 2015-10-16

**Authors:** Sofie E. De Meyer, Matthew Parker, Peter Van Berkum, Rui Tian, Rekha Seshadri, T.B.K. Reddy, Victor Markowitz, Natalia Ivanova, Amrita Pati, Tanja Woyke, Nikos Kyrpides, John Howieson, Wayne Reeve

**Affiliations:** Centre for Rhizobium Studies, Murdoch University, Murdoch, Western Australia; Binghamton University, State University of New York, New York, USA; Soybean Genomics and improvement laboratory Bldg 006, BARC-West USDA ARS 10300 Baltimore Blvd, Beltsville, MD 20705 USA; DOE Joint Genome Institute, Walnut Creek, CA USA; Biological Data Management and Technology Center, Lawrence Berkeley National Laboratory, Berkeley, CA USA; Department of Biological Sciences, King Abdulaziz University, Jeddah, Saudi Arabia

**Keywords:** Root-nodule bacteria, Nitrogen fixation, *Betaproteobacteria*, Texas, *Mimosa asperata*, GEBA-RNB

## Abstract

*Cupriavidus* sp. strain AMP6 is an aerobic, motile, Gram-negative, non-spore-forming rod that was isolated from a root nodule of *Mimosa asperata* collected in Santa Ana National Wildlife Refuge, Texas, in 2005. *Mimosa asperata* is the only legume described so far to exclusively associates with *Cupriavidus* symbionts. Moreover, strain AMP6 represents an early-diverging lineage within the symbiotic *Cupriavidus* group and has the capacity to develop an effective nitrogen-fixing symbiosis with three other species of *Mimosa*. Therefore, the genome of *Cupriavidus* sp. strain AMP6 enables comparative analyses of symbiotic trait evolution in this genus and here we describe the general features, together with sequence and annotation. The 7,579,563 bp high-quality permanent draft genome is arranged in 260 scaffolds of 262 contigs, contains 7,033 protein-coding genes and 97 RNA-only encoding genes, and is part of the GEBA-RNB project proposal.

## Introduction

*Cupriavidus* is one of two known genera of *Betaproteobacteria* that include legume root-nodule symbionts [1]. The other genus, *Burkholderia*, has multiple species associated with diverse legume host plants indigenous to North and South America, South Africa and Australia [2–8]. *Cupriavidus*, by contrast, has only been isolated from four species in two legume genera in the tribe Mimoseae (*Mimosa**, Parapiptadenia*), at a few locations in the native geographic ranges of their host plants (south Texas, the Caribbean, central America, French Guiana, and Uruguay; [2, 9–12]). However, both *Cupriavidus* and *Burkholderia* have now spread to many new regions along with species of *Mimosa* that are invasive weeds [10, 13–17]. In South America, *Cupriavidus* was uncommon in French Guiana and Uruguay (3-10 % of nodule isolates; [9, 11]), and was not detected at all in extensive surveys of *Mimosa* in central Brazil [5, 6]. However, it has been isolated from two cultivated legumes in Minas Gerais, Brazil [18]. This suggests that further surveys in South America may discover additional wild legume hosts that utilize *Cupriavidus* symbionts.

The only legume studied to date that is exclusively associated with *Cupriavidus* nodule symbionts is *Mimosa asperata*, from which *Cupriavidus* strain AMP6 was isolated in 2005 [12]. The range of *M. asperata* is centered in Mexico and extends slightly into south Texas, Cuba, and northern Central America [19]. Based on both housekeeping loci and symbiotic loci, strain AMP6 represents an early-diverging lineage of nodule-symbiotic *Cupriavidus* [10, 12], whose genome may provide insights about how legume nodule symbiosis became established in this group.

Strain AMP6 was collected at the Santa Ana National Wildlife Refuge in Hidalgo County, Texas. *Cupriavidus* nodule bacteria resembling strain AMP6 are currently known only from *M. asperata* populations in the lower Rio Grande valley of Texas, and have not been detected in surveys of *Mimosa* species in other geographic locations [2, 9–11]. Nevertheless, inoculation tests have indicated that *Cupriavidus* strain AMP6 has the capacity to develop an effective nitrogen-fixing symbiosis with three other species of *Mimosa* [12]. *M. asperata* occurs mainly along the margins of seasonally flooded wetlands [20], a habitat characterized by heavy silt/clay soils with neutral to moderately alkaline pH (pH 7.0 - 8.4; [21]).

The first completed genome for a betaproteobacterial legume symbiont was that of *Cupriavidus taiwanensis*LMG 19424^T^ [22]. Here we provide an analysis of the high-quality permanent draft genome sequence of *Cupriavidus* strain AMP6, enabling comparative analyses of symbiotic trait evolution in this genus.

### Organism information

#### Classification and features

**Fig. 1:**
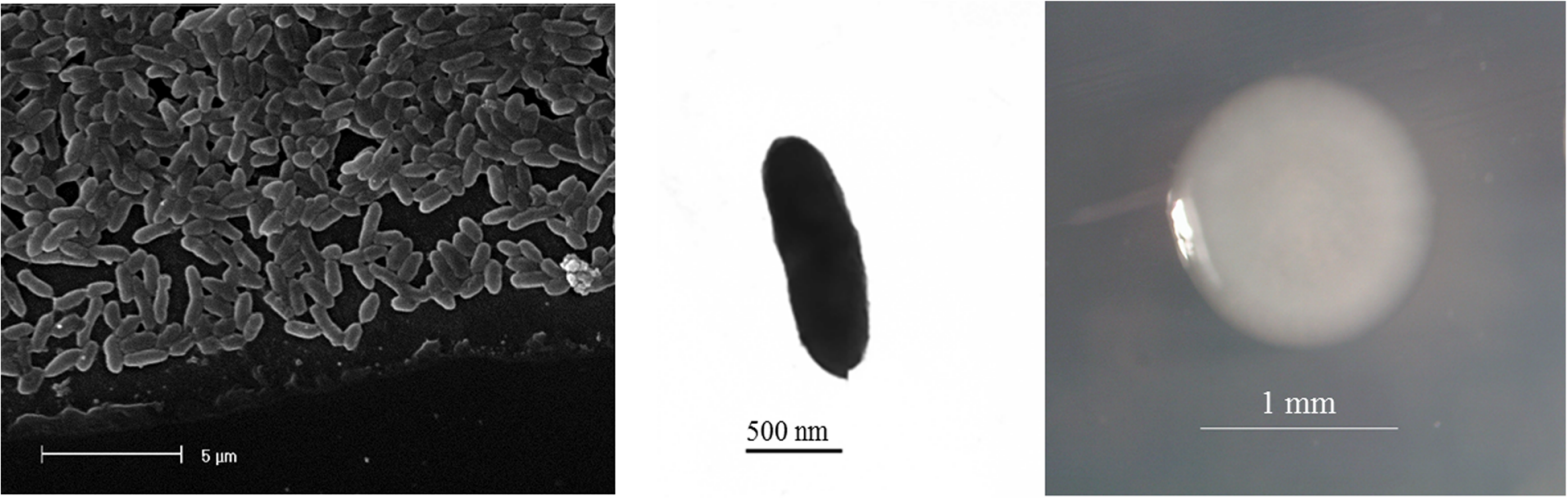
Images of *Cupriavidus* sp. strain AMP6 using scanning (Left) and transmission (Center) electron microscopy and the appearance of colony morphology on solid media (Right)

*Cupriavidus* sp. strain AMP6 is a motile, Gram-negative, non-spore-forming rod (Fig. 1 Left, Center) in the order *Burkholderiales* of the class *Betaproteobacteria*. The rod-shaped form varies in size with dimensions of 0.4-0.6 μm in width and 1.2-1.7 μm in length (Fig. 1 Left). It is fast growing, forming 1.2-1.6 mm diameter colonies after 24 h when grown on YMA [23] at 28 °C. Colonies on YMA are white-opaque, slightly domed, moderately mucoid with smooth margins (Fig. 1 Right).

**Fig. 2:**
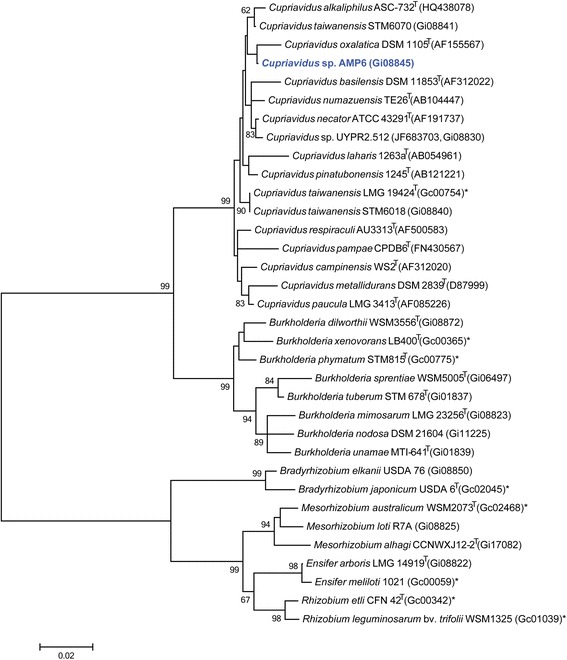
Phylogenetic tree highlighting the position of *Cupriavidus* sp. strain AMP6 (shown in blue print) relative to other type and non-type strains in the *Cupriavidus* genus using a 1,024 bp internal region of the 16S rRNA gene. Several Alpha-rhizobia sequences were used as an outgroup. All sites were informative and there were no gap-containing sites. Phylogenetic analyses were performed using MEGA, version 5.05 [46]. The tree was build using the maximum likelihood method with the General Time Reversible model. Bootstrap analysis with 500 replicates was performed to assess the support of the clusters. Type strains are indicated with a superscript T. Strains with a genome sequencing project registered in GOLD [30] have the GOLD ID mentioned after the strain number, otherwise the NCBI accession number is provided. Finished genomes are designated with an asterisk

Figure 2 shows the phylogenetic relationship of *Cupriavidus* sp. strain AMP6 in a 16S rRNA gene sequence based tree. This strain is phylogenetically most related to *Cupriavidus taiwanensis*LMG 19424^T^, *Cupriavidus alkaliphilus*ASC-732^T^ and *Cupriavidus necator* N-1^T^ (deposited as ATCC43291^T^) with sequence identities to the AMP6 16S rRNA gene sequence of 99.11 %, 99.04 % and 98.69 %, respectively, as determined using the EzTaxon-e server [24]. *Cupriavidus taiwanensis*LMG 19424^T^ is a plant symbiont and was isolated from root nodules of *Mimosa pudica* collected from three fields at Ping-Tung Country in the southern part of Taiwan [25]. Both ASC-732^T^ and N-1^T^ are soil bacteria that are not able to nodulate or fix nitrogen with legumes [26, 27]. Minimum Information about the Genome Sequence (MIGS) [28] of AMP6 is provided in Table 1.

**Table 1:** Classification and general features of *Cupriavidus* sp. strain AMP6 in accordance with the MIGS recommendations [28] published by the Genome Standards Consortium [47]

MIGS ID	Property	Term	Evidence code
	Classification	Domain *Bacteria*	TAS [48]
		Phylum *Proteobacteria*	TAS [49, 50]
		Class *Betaproteobacteria*	TAS [51]
		Order *Burkholderiales*	TAS [52]
		Family *Burkholderiaceae*	TAS [53]
		Genus *Cupriavidus*	TAS [54]
		Species *Cupriavidus* sp.	TAS [12]
		(Type) strain AMP6	TAS [12]
	Gram stain	Negative	TAS [54]
	Cell shape	Rod	IDA
	Motility	Motile	IDA
	Sporulation	Non-sporulating	TAS [54]
	Temperature range	Mesophile	TAS [54]
	Optimum temperature	28 °C	IDA
	pH range; Optimum	Not reported	
	Carbon source	Not reported	
MIGS-6	Habitat	Soil, root nodule on host	IDA
MIGS-6.3	Salinity	Not reported	
MIGS-22	Oxygen requirement	Aerobic	IDA
MIGS-15	Biotic relationship	Symbiotic	IDA
MIGS-14	Pathogenicity	Non-pathogenic	NAS
MIGS-4	Geographic location	Texas, USA	TAS [12]
MIGS-5	Nodule collection date	2005	TAS [12]
MIGS-4.1	Longitude	−98.138	TAS [12]
MIGS-4.2	Latitude	26.0794	TAS [12]
MIGS-4.4	Altitude	30 m	IDA

Evidence codes – IDA: Inferred from Direct Assay; TAS: Traceable Author Statement (i.e., a direct report exists in the literature); NAS: Non-traceable Author Statement (i.e., not directly observed for the living, isolated sample, but based on a generally accepted property for the species, or anecdotal evidence). These evidence codes are from the Gene Ontology project [55]

### Symbiotaxonomy

*Cupriavidus* sp. strain AMP6 was isolated from *Mimosa asperata* nodules collected at the Santa Ana National Wildlife Refuge in Hidalgo County, Texas [12]. *Cupriavidus* sp. strain AMP6 was assessed for nodulation and nitrogen fixation on five mimosa species, including *M. pigra, M. pudica, M. invisia, M. strigillosa* and *M. quadrivalvis* [12]. Strain AMP6 could nodulate all hosts apart from *M. quadrivalvis* [12]. Additional acetylene reduction assays provided information on the nitrogenase activity of strain AMP6 on those hosts. These test showed substantial nitrogenase activity with *M. pudica* and *M. invisia* but only a small amount with *M. pigra* [12]. The absence of nodule nitrogenase activity was also observed for *M. strigillosa* and *M. quadrivalvis* [12].

### Genome sequencing information

#### Genome project history

This organism was selected for sequencing on the basis of its environmental and agricultural relevance to issues in global carbon cycling, alternative energy production, and biogeochemical importance, and is part of the Genomic Encyclopedia of Bacteria and Archaea, The Root Nodulating Bacteria chapter project at the U.S. Department of Energy, Joint Genome Institute [29]. The genome project is deposited in the Genomes OnLine Database [30] and the high-quality permanent draft genome sequence in IMG [31]. Sequencing, finishing and annotation were performed by the JGI using state of the art sequencing technology [32]. A summary of the project information is shown in Table 2.

**Table 2:** Genome sequencing project information for *Cupriavidus* sp. strain AMP6

MIGS ID	Property	Term
MIGS-31	Finishing quality	High-quality permanent draft
MIGS-28	Libraries used	Illumina Std PE
MIGS-29	Sequencing platforms	Illumina HiSeq 2000
MIGS-31.2	Fold coverage	117.0x Illumina
MIGS-30	Assemblers	Velvet 1.1.04, ALLPATHS V.r42328
MIGS-32	Gene calling methods	Prodigal 1.4
	Locus Tag	K309
	Genbank ID	AUFE00000000
	Genbank Date of Release	December 12, 2013
	GOLD ID	Gp0009812
	BIOPROJECT	PRJNA195776
MIGS-13	Source Material Identifier	AMP6
	Project relevance	Symbiotic N_2_fixation, agriculture

### Growth conditions and genomic DNA preparation

*Cupriavidus* sp. strain AMP6 was grown on YMA solid medium [23] for 3 days, a single colony was selected and used to inoculate 5 ml TY broth medium. The culture was grown for 48 h on a gyratory shaker (200 rpm) at 28 °C. Subsequently 1 ml was used to inoculate 60 ml TY broth medium and grown on a gyratory shaker (200 rpm) at 28 °C until OD 0.6 was reached. DNA was isolated from 60 mL of cells using a CTAB bacterial genomic DNA isolation method [33]. Final concentration of the DNA was 0.6 mg/ml.

### Genome sequencing and assembly

The genome of *Cupriavidus* sp. AMP6 was generated at the DOE Joint genome Institute [32]. An Illumina Std shotgun library was constructed and sequenced using the Illumina HiSeq 2000 platform which generated 15,823,344 reads totaling 2,373.5 Mbp. All general aspects of library construction and sequencing performed at the JGI can be found at the JGI web site [34]. All raw Illumina sequence data was passed through DUK, a filtering program developed at JGI, which removes known Illumina sequencing and library preparation artifacts (Mingkun L, Copeland A, Han J. unpublished). Following steps were then performed for assembly: (1) filtered Illumina reads were assembled using Velvet (version 1.1.04) [35] (2) 1–3 Kbp simulated paired end reads were created from Velvet contigs using wgsim [36] (3) Illumina reads were assembled with simulated read pairs using Allpaths–LG (version r42328) [37]. Parameters for assembly steps were: 1) Velvet (velveth: 63 –shortPaired and velvetg: −very clean yes –exportFiltered yes –min contig lgth 500 –scaffolding no–cov cutoff 10) 2) wgsim (−e 0 –1 100 –2 100 –r 0 –R 0 –X 0) 3) Allpaths–LG (PrepareAllpathsInputs: PHRED 64 = 1 PLOIDY = 1 FRAG COVERAGE = 125 JUMP COVERAGE = 25 LONG JUMP COV = 50, RunAllpathsLG: THREADS = 8 RUN = std shredpairs TARGETS = standard VAPI WARN ONLY = True OVERWRITE = True). The final draft assembly contained 262 contigs in 260 scaffolds. The total size of the genome is 7.6 Mbp and the final assembly is based on 886.3 Mbp of Illumina data, which provides an average of 117.0× coverage of the genome.

### Genome annotation

Genes were identified using Prodigal [38], as part of the DOE-JGI genome annotation pipeline [39, 40] followed by a round of manual curation using GenePRIMP [41] for finished genomes and Draft genomes in fewer than 10 scaffolds. The predicted CDSs were translated and used to search the NCBI non-redundant database, UniProt, TIGRFam, Pfam, KEGG, COG, and InterPro databases. The tRNAScanSE tool [42] was used to find tRNA genes, whereas ribosomal RNA genes were found by searches against models of the ribosomal RNA genes built from SILVA [43]. Other non–coding RNAs such as the RNA components of the protein secretion complex and the RNase P were identified by searching the genome for the corresponding Rfam profiles using INFERNAL [44]. Additional gene prediction analysis and manual functional annotation was performed within the Integrated Microbial Genomes-Expert Review (IMG-ER) system [45] developed by the Joint Genome Institute, Walnut Creek, CA, USA.

### Genome properties

The genome is 7,579,563 nucleotides with 65.46 % GC content (Table 3) and comprised of 260 scaffolds and 262 contigs. From a total of 7,130 genes, 7,033 were protein encoding and 97 RNA only encoding genes. The majority of genes (80.24 %) were assigned a putative function whilst the remaining genes were annotated as hypothetical. The distribution of genes into COG functional categories is presented in Table 4.

**Table 3:** Genome statistics for *Cupriavidus* sp. AMP6

Attribute	Value	% of Total
Genome size (bp)	7,579,563	100.00
DNA coding (bp)	6,545,489	86.36
DNA G + C (bp)	4,961,426	65.46
DNA scaffolds	260	100.00
Total genes	7,130	100.00
Protein-coding genes	7,033	98.64
RNA genes	97	1.36
Pseudo genes	0	0.00
Genes in internal clusters	538	7.55
Genes with function prediction	5,721	80.24
Genes assigned to COGs	4,791	67.19
Genes with Pfam domains	5,837	81.87
Genes with signal peptides	681	9.55
Genes with transmembrane helices	1,477	20.72
CRISPR repeats	1

**Table 4:** Number of genes associated with general COG functional categories

Code	Value	% age	COG Category
J	182	3.37	Translation, ribosomal structure and biogenesis
A	1	0.02	RNA processing and modification
K	527	9.76	Transcription
L	188	3.48	Replication, recombination and repair
B	4	0.07	Chromatin structure and dynamics
D	32	0.59	Cell cycle control, Cell division, chromosome partitioning
V	59	1.09	Defense mechanisms
T	210	3.89	Signal transduction mechanisms
M	275	5.09	Cell wall/membrane/envelope biogenesis
N	96	1.78	Cell motility
U	119	2.20	Intracellular trafficking, secretion, and vesicular transport
O	164	3.04	Posttranslational modification, protein turnover, chaperones
C	447	8.28	Energy production and conversion
G	256	4.74	Carbohydrate transport and metabolism
E	501	9.28	Amino acid transport and metabolism
F	90	1.67	Nucleotide transport and metabolism
H	185	3.43	Coenzyme transport and metabolism
I	344	6.37	Lipid transport and metabolism
P	272	5.04	Inorganic ion transport and metabolism
Q	235	4.35	Secondary metabolite biosynthesis, transport and catabolism
R	659	12.21	General function prediction only
S	552	10.23	Function unknown
-	2339	32.81	Not in COGS

The total is based on the total number of protein coding genes in the genome

## Conclusion

*Cupriavidus* sp. AMP6 belongs to a group of Beta-rhizobia isolated from *Mimosa asperata*. Phylogenetic analysis revealed that AMP6 is most closely related to *Cupriavidus taiwanensis*LMG 19424^T^, which was isolated from *Mimosa pudica**,* and is able to nodulate and fix nitrogen in association with several *Mimosa* species [13]. In total five *Cupriavidus* strains (AMP6, LMG 19424^T^, STM6018, STM6070 and UYPR2.512), which can form a symbiotic association have now been sequenced. A comparison of these strains reveals that AMP6 has the second largest genome (7.6 Mbp), with the highest KOG count (1398) and the second lowest GC (65.46 %) and signal peptide (9.55 %) percentages in this group. All of these genomes share the nitrogenase-RXN MetaCyc pathway characterized by the multiprotein nitrogenase complex. Out of five *Cupriavidus* strains (AMP6, LMG 19424^T^, STM6018, STM6070 and UYPR2.512), which contain the N-fixation pathway, only *Cupriavidus* sp. AMP6 has been shown to fix effectively with *Mimosa asperata*. The genome attributes of *Cupriavidus* sp. AMP6, in conjunction with other *Cupriavidus* genomes, will be important for ongoing molecular analysis of the plant microbe interactions required for the establishment of *Mimosa* symbioses.

## Abbreviations

GEBA-RNB: Genomic Encyclopedia of Bacteria and Archaea – Root Nodule Bacteria
JGI: Joint Genome Institute
TY: Trypton Yeast
CTAB: Cetyl trimethyl ammonium bromide
WSM: Western Australian Soil Microbiology
